# Post‐operative joint stiffness after Bereiter trochleoplasty does not affect 2‐year improvement in patient‐reported outcomes. A prospective cohort study of 374 Bereiter trochleoplasties

**DOI:** 10.1002/ksa.12645

**Published:** 2025-03-07

**Authors:** Christian Dippmann, Christos Soleas, Anke Simone Rechter, Volkert Siersma, Kristoffer Weisskirchner Barfod, Peter Lavard

**Affiliations:** ^1^ Section for Sports Traumatology M51, Department of Orthopedic Surgery Copenhagen University Hospital Bispebjerg Copenhagen Denmark; ^2^ Department of Orthopedic Surgery Zealand University Hospital, Nykøbing F. Nykøbing Falster Denmark; ^3^ The Research Unit for General Practice and Section of General Practice, Department of Public Health University of Copenhagen Copenhagen Denmark

**Keywords:** Bereiter trochleoplasty, patello‐femoral instability, post‐operative joint stiffness, trochlar dysplasia complication

## Abstract

**Purpose:**

Bereiter trochleoplasty (TP) is a well‐described procedure to address trochlear dysplasia (TD). Post‐operative joint stiffness with reduced range of motion (ROM) is a common complication usually requiring arthroscopically assisted manipulation (AAM) with the removal of adhesions and scar tissue. Inferior clinical outcomes after TP have been reported for patients with subsequent surgery. We hypothesised that a 2‐year improvement in patient‐reported outcomes would be lower in patients treated with AAM.

**Methods:**

This was a retrospective cohort study of prospectively collected data comparing subgroups of patients with and without post‐operative joint stiffness from a consecutive cohort of 374 knees with high‐grade TD who underwent TP according to the Copenhagen patello‐femoral instability (PFI) algorithm. All patients received supervised training exercises led by a physiotherapist. At 3‐month follow‐up, patients with an extension deficit >10° and/or flexion <120° were diagnosed with post‐operative joint stiffness and treated with AAM. Outcomes were mean differences from baseline in Kujala, Knee injury and Osteoarthritis Outcome Score (KOOS) and Lysholm scores 1 and 2 years after surgery.

**Results:**

Forty‐nine (38 females, 11 males) of the 374 knees (12%) had post‐operative joint stiffness and underwent AAM. Nine patients underwent subsequent AAMs. Full extension and flexion >135° were achieved in 37 out of 49 cases (75%). In 11 cases, flexion remained reduced, while data on ROM could not be retrieved in one case. While both patients with and without AAM showed clinically relevant improvements in the Kujala, KOOS and Lysholm scores, no statistically significant between‐group differences were seen in these improvements.

**Conclusions:**

Post‐operative joint stiffness was a common complication after Bereiter TP following the Copenhagen PFI algorithm. Twenty‐five per cent of the AAM patients, or 3% of the study population, did not regain full ROM. We did not find that post‐operative joint stiffness was associated with inferior improvements in patient‐reported outcomes 1 and 2 years after surgery.

**Level of Evidence:**

Level IV, a retrospective cohort study.

**Abbreviations**
95% CI: 95% confidence interval
AAM: arthroscopically assisted manipulation
ET: Elmslie–Trillat
IKDC: International Knee Documentation Committee
KOOS: Knee injury and Osteoarthritis Outcome Score
LTI: lateral trochlear inclination
MACI: matrix‐induced autologous chondrocyte implantation
MCID: minimal clinically important difference
MDE: minimal detectable effect
MPFL‐R: medial patella‐femoral ligament reconstruction
PFI: patello‐femoral instability
PROM: patient‐reported outcome measure
ROM: range of motion
SD: standard deviation
TD: trochlear dysplasia
TP: trochleoplasty
TT‐TG: tibial tuberosity trochlea groove

## INTRODUCTION

Trochlea dysplasia (TD) is the most important, single risk factor for recurrent patello‐femoral instability (PFI) and is usually treated with open trochleoplasty (TP) [4]. As it is advantageous to address all relevant morphologic risk factors in a single procedure, TP is often combined with other osseous and/or soft‐tissue procedures [23]. At this institution, all patients with PFI due to TD have been treated following the Copenhagen PFI algorithm, including a Bereiter thin flap TP, since 2011 [10].

Major complications after Bereiter TP are rare, but subsequent surgery is reported in 27%–56% of cases due to metal removal, adhesion, pain, recurrent instability and joint stiffness [8, 12, 14]. Post‐operative joint stiffness leading to arthroscopically assisted manipulation (AAM) is reported in 4.3%–19% of cases [5, 12, 22]. The cause of post‐operative joint stiffness is unknown, potentially involving intrinsic (e.g., genetic) and extrinsic (e.g., surgical trauma, pain, rehabilitation and compliance) risk factors [26]. As stated by Carstensen et al. [2], post‐operative joint stiffness may negatively influence the patient‐reported outcome after Bereiter TP with a prolonged overall recovery time, reduced range of motion (ROM) and additional risk of complications [12].

The purpose of this study was to investigate the 2‐year improvements in outcomes in patients with and without post‐operative joint stiffness after Bereiter TP for high‐grade TD in a cohort of 374 consecutive patients treated according to the Copenhagen PFI algorithm from 2011 to 2021. It was hypothesised that the improvements in outcome would be lower in patients with post‐operative joint stiffness treated with AAM.

## MATERIALS AND METHODS

This was a retrospective cohort study of prospectively collected data, comparing subgroups of patients with and without post‐operative joint stiffness after Bereiter TP.

### Patient cohort

All high‐grade TD patients treated with Bereiter TP according to the Copenhagen PFI algorithm were registered in the database and followed prospectively after giving written consent. Patients registered from August 2010 to September 2021 were enroled in the present study.

### The Copenhagen PFI algorithm

**Figure 1:**
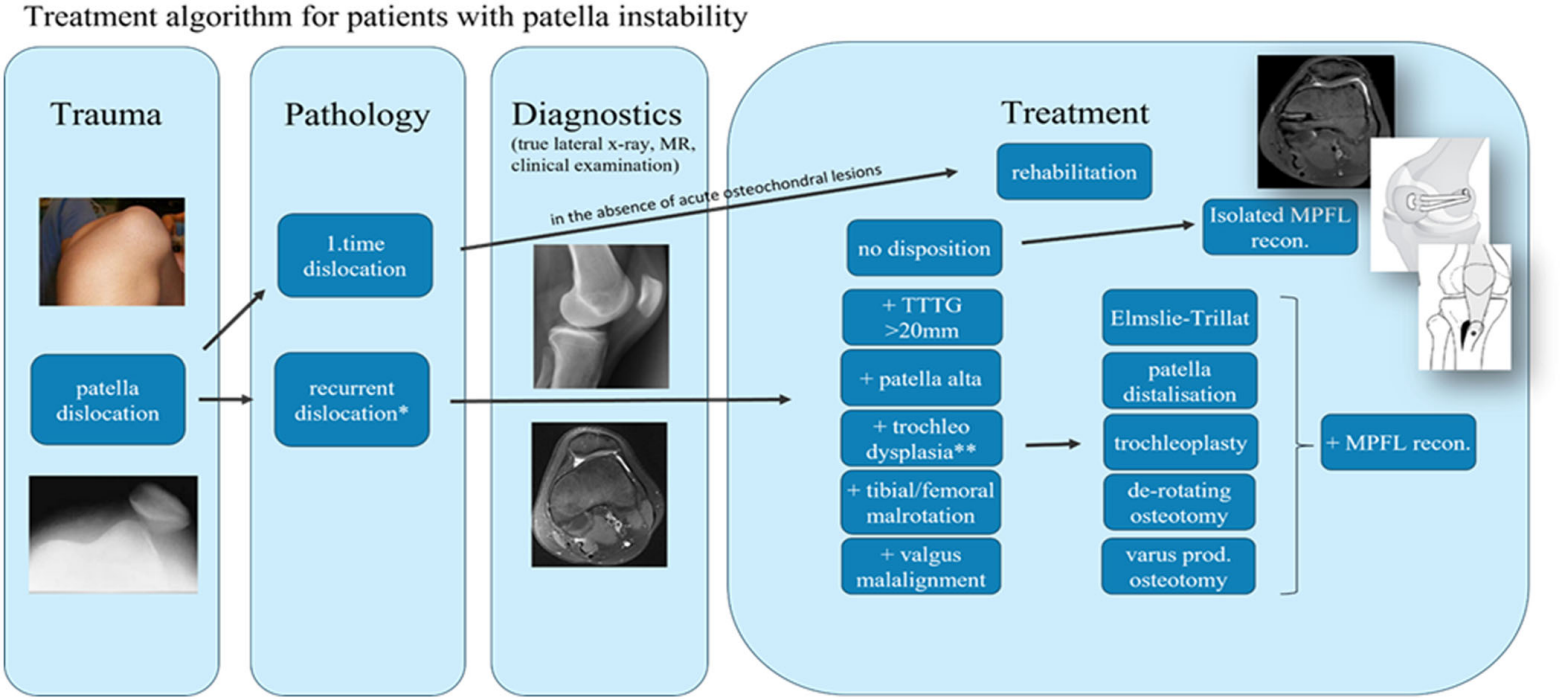
Treatment algorithm for patients with patellar instability. Patients with recurrent dislocations or permanent/habitual patellar dislocation are examined clinically and by true lateral radiograph, MRI, ±CT scan and long‐standing radiograph to identify all relevant predisposing factors. The following cut‐off values are used: TT‐TG > 20 mm on MRI scan for medialisation of the TT (Elmslie–Trillat), CD index >1.2 for distalisation of the TT, LTI < 11° for trochleoplasty, tibial torsion >40° and/or femoral torsion >35° for de‐rotating osteotomy and valgus angle >10° for varus producing osteotomy [5]. CD index, Caton–Deschamps index; CT, computed tomography; MRI, magnetic resonance imaging; TT‐TG, tibial tuberosity trochlea groove.

The Copenhagen PFI algorithm (Figure 1) has been used with minor alterations since August 2009 [5]. Following the algorithm, all patients with high‐grade TD (Dejour B and D), a lateral trochlear inclination (LTI) < 11° and more than one patella dislocation were offered TP and reconstruction of the medial patello‐femoral ligament reconstruction (MPFL‐R) as a minimum. Additional procedures such as medialisation and/or distalisation of the tibial tuberosity (TT), varus‐producing osteotomy and/or derotation osteotomy were performed after a thorough clinical and paraclinical analysis, including true lateral X‐ray, magnetic resonance imaging scan and clinical tests (apprehension, j‐sign, patella lateralisation, rotational alignment, etc.). The details of the surgical indication and technique, as well as the 5‐year outcome, have been published earlier [5].

### Surgical technique and post‐operative care

TP was performed using the Bereiter thin flap technique in combination with an MPFL reconstruction with a gracilis autograft as the first choice [1, 5]. The patients were discharged the day after surgery. Post‐operatively, all patients were partially immobilised in a hinged knee brace locked in 40° of flexion for the first 2 weeks, followed by free ROM for the next 4 weeks. The patients were allowed full weight‐bearing from Week 2.

### Rehabilitation

The patients were referred to supervised rehabilitation focusing on full extension and flexion. In patients with clinical signs of a post‐operative stiff knee within the first 6 weeks after surgery, rehabilitation was intensified, and intraarticular injection with glucocorticoids was considered.

### AAM

AAM was scheduled at the 3‐month follow‐up consultation if patients presented reduced extension (>5°) or flexion (<90°). Patients who underwent AAM were hospitalised for at least 2 days for intensive, supervised rehabilitation under oral and regional pain management. All patients received a regional nervus saphenous block [16]. Between the rehabilitation sessions, the operated knee was passively mobilised using a Kinetec Knee CPM Unit®. If considered necessary, the Kinetec unit was lent to the patient for the next 2 weeks, while being followed by their physiotherapist on a regular basis. ROM was considered normalised when the extension deficit was <5° and flexion >135°.

### Data collection

Data were collected prospectively at baseline and 1 and 2 years after TP. Sex, age, radiological data and information regarding previous surgery were collected from the patients' electronic records (Epic®). Additionally, all information about patients who underwent AAM were analysed regarding ROM, time from TP to AAM and clinical outcome after AAM. The ROM for patients with normal post‐operative ROM and no AAM was only reported when exceeding normal values. Institutional review board approval for the present study was achieved from the data registration committee (P‐2020‐573) and the ethical committee (Journal‐no: H‐20031969) of the capital region of Denmark.

### Outcomes

Patients completed three patient‐related outcome measures (PROMs) prior to surgery and at follow‐up: the Kujala score [11], the Knee injury and Osteoarthritis Outcome Score (KOOS, consisting of five separate domains) [21] and the Lysholm score [25]. Each of the scores and domains scores from 0 (*worst score*) to 100 (*best score*) [7, 17, 18, 28].

### Statistical analysis

Mean differences from baseline for the PROMs and their 95% confidence intervals (95% CIs) were estimated in mixed linear regression models featuring a patient random effect to adjust the variance for repeated observations on the same person. These estimates were adjusted for patient age and sex and whether subsequent surgery other than AAM was performed after TP. Mean differences were estimated separately for patients with and without subsequent AAM, and interaction between follow‐up time and the AAM indicator evaluated differences between these two groups. Analyses were performed in SAS PROC MIXED, SAS version 9.4.

Especially in the group with AAM, the number of patients may be too small to detect changes in the PROMs that are relevant to the patient. Lacking minimal clinically important differences for the PROMs used in this study for patients with patellofemoral instability, a change of 50% of the preoperative standard deviation (SD), that is, 0.5 SD, between preoperative and post‐operative scores was taken as a rough estimate of the relevant threshold for patient‐perceived change [15]. For each analysis, we determined the minimal detectable effect with power 80% (0.8MDE), that is, the size of the change that can be found with 80% probability with 5% statistical significance with the available data, calculated by multiplying half the width of the corresponding 95% CI with a factor *ϕ* = 1.43, see [13]. Hence, 0.8MDE > 0.5 SD indicates insufficient power to detect a clinically relevant effect. However, note that even when power is insufficient according to the above criterion, a statistically significant improvement larger than 0.5 SD indicates a clinically relevant improvement.

## RESULTS

**Figure 2:**
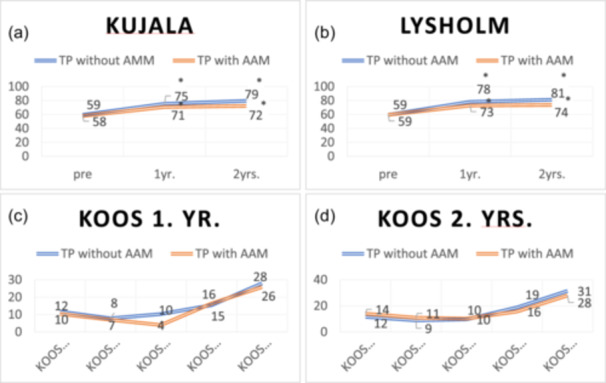
Significant improvement 1 and 2 years after Bereiter TP can be seen without detectable difference between patients undergoing subsequent arthroscopically assisted manipulation (AAM). (a) and (b) present the average Kujala and Lysholm scores pre‐operatively, 1 and 2 years after surgery, while (c) and (d) illustrate the difference in mean improvement in KOOS (96% CI) from baseline after 1 and 2 years. Ci, confidence interval; KOOS, Knee injury and Osteoarthritis Outcome Score; TP, trochleoplasty.

During the study period, 374 knees underwent Bereiter TP, among which 49 (12%) were diagnosed with post‐operative joint stiffness and underwent AAM (Table 1). Full extension and flexion >135° were achieved in 37 out of 49 cases (75%). In 11 cases, flexion remained reduced, while data on ROM could not be retrieved in one case. Table 2 shows that improvements for KOOS symptoms did not reach the 0.5 SD threshold in the group without AAM after 1 and 2 years and in the group with AAM after 1 year. Also, for the group with AAM, KOOS ADL did not reach the 0.5 SD threshold after 1 year. All other improvements were estimated over the 0.5 SD threshold and deemed clinically relevant. Furthermore, comparison of 0.8MDE and 0.5 SD indicated that the analyses were underpowered in the AAM group for KOOS sport, KOOS quality of life (QoL), and Lysholm after 1 year, and KOOS sport, KOOS ADL, KOOS QoL and Lysholm after 2 years. Only for KOOS ADL after 1 year, the improvement in the AAM group was not statistically significant. No statistically significant differences in mean improvement from baseline were seen in the PROMs between patients with and without post‐operative joint stiffness at 1 and 2 years of follow‐up (Figure 2).

**Table 1:** Patient characteristics, procedures performed and previous surgery.

Patient characteristics			
		TP with AAM	TP without AAM
Cases		49	325
Side	Left	23	173
	Right	26	152
	Bilateral	4	35
Age	22 years	24 years [15–43 years]	22 years [12–47 years]
Sex	Female	38 (76%)	229 (71%)
	Male	11 (34%)	96 (29%)
BMI		23.5 [16–38]	24.9 [14.1–43.3]
Previous surgery		8 (16%)	159 (49%)
	Soft tissue procedures (medial reefing, etc.)	2	25
	MPFL‐R + Elmslie–Trillat (ET)	0	18
	MPFL‐R	2	16
	ET	2	11
	Knee arthroscopy	1	9
	MACI	1	0
Procedures performed	Isolated trochleoplasty (TP) + MPFL‐R	22 (45%)	167 (52%)
	TP + MPFL‐R with ET	15 (31%)	88 (27%)
	TP + MPFL‐R with ET and TT distalisation	9 (18%)	50 (15%)
	TP + MPFL‐R with TT distalisation	3 (6%)	12 (4%)
	Others		8 (2%)
	Concomitant procedures		
	Vastus plasty	1 (2%)	4 (1%)
	De‐rotating distal femur osteotomy	1 (2%)	2 (1%)
	Varus producing distal femur osteotomy	0	3 (0.3%)
	Osteochondral allograft transplantation	0	1 (0.3%)
AAM	Average time to AAM (weeks)	12.1 [4–24]	
	Attempts of AAM needed		
	Single	40	
	Two	7	
	Three	2	
	Full ROM achieved *n* (%)	37 (75%)	
	(flexion > 135°, neutral extension)		

Abbreviations: AAM, arthroscopically assisted manipulation; MACI, matrix‐induced autologous chondrocyte implantation; MPFL‐R, medial patellofemoral ligament reconstruction; TT, tibial tuberosity.

**Table 2:** The table presents the difference in mean improvement (95% CI) from baseline to 1 and 2 years after surgery in relation to 80% MDE.

			1 Year of follow‐up				2 Years of follow‐up				
		0.5SD^a^	*N*	Mean diff (95% CI)	0.5SD^b^	0.8MDE	*N*	Mean diff (95% CI)	0.5SD^c^	0.8MDE	Sign.
TP without AAM	Kujala	8.7	235	15.6 (13.3–17.8)	7.7	3.3	205	19.0 (16.6–21.4)	8.3	3.4	0.6890
	KOOS Pain	9.5	237	11.7 (9.4–14.0)	7.2	3.3	203	11.5 (9.1–14.0)	7.9	3.5	0.5171
	KOOS Symp	9.3	237	7.8 (5.4–10.1)	7.7	3.3	201	8.9 (6.4–11.3)	7.5	3.5	0.6824
	KOOS ADL	9.9	237	10.3 (7.7–12.9)	6.8	3.7	202	9.7 (7.0–12.4)	8.0	3.9	0.1049
	KOOS Sport	14.3	235	15.4 (11.5–19.2)	13.7	5.5	202	18.7 (14.7–22.7)	14.3	5.7	0.7429
	KOOS QoL	8.5	236	28.0 (24.9–31.0)	11.4	4.4	201	31.5 (28.2–34.7)	12.1	4.6	0.7493
	Lysholm	9.6	165	18.0 (14.9–21.0)	8.3	4.4	164	20.3 (17.2–23.4)	8.1	4.4	0.8285
TP with AAM	Kujala	8.7	33	13.0 (7.2–18.8)	10.1	8.3	30	16.8 (10.7–22.9)	8.9	8.7	
	KOOS Pain	9.5	34	10.4 (4.1–16.6)	10.5	8.9	30	14.0 (7.4–20.5)	9.9	9.3	
	KOOS Symp	9.3	34	7.0 (0.8–13.1)	7.9	8.8	30	10.9 (4.4–17.4)	7.5	9.3	
	KOOS ADL	9.9	34	4.0 (−2.8 to 10.8)	12.4	9.7	30	10.2 (3.1–17.4)	9.0	10.2	
	KOOS Sport	14.3	34	16.4 (6.2–26.5)	15.6	14.5	30	15.7 (5.0–26.3)	16.9	15.2	
	KOOS QoL	8.5	34	25.8 (17.8–33.9)	10.7	11.5	30	28.0 (19.5–36.4)	11.5	12.1	
	Lysholm	9.6	28	15.7 (7.7–23.6)	10.1	11.4	25	17.7 (9.4–26.1)	8.0	11.9	

*Note*: All PROM scores show significant improvement after 1 and 2 years. However, KOOS symptoms and KOOS QoL, and Lysholm after 1 year, and KOOS symptoms, KOOS ADL, KOOS QoL and Lysholm after 2 years may not have enough power to be concluded on as 0.8 MDE ≥ 0.5 SD is indicative of insufficient power.

Abbreviations: AAM, arthroscopically assisted manipulation; ADL, activities of daily living; KOOS, Knee injury and Osteoarthritis Outcome Score; MCID, minimal clinically important difference; MDE, minimal detectable effect; QoL, quality of life; SD, standard deviation.

^a^ Assessment of the MCID by half of the standard deviation (0.5SD) of the outcome at baseline [13].

^b^ Minimal detectable effect with power 80% (0.8MDE): the size of the effect that can be found with 80% probability and 5% statistical significance with the available data, calculated by multiplying half the width of the 95% CI with *ϕ *= 1.43, see [11].

^c^ *p*‐Value of a test whether the mean differences between patients with AAM and without AAM are different, jointly for 1 and 2 years of follow‐up.

## DISCUSSION

The most important finding of the present study was that patients undergoing AAM due to post‐operative joint stiffness after Bereiter TP did not report inferior improvements 1 and 2 years after surgery compared to patients without AAM.

Post‐operative joint stiffness is a common complication following TP. The overall risk varies depending on the TP technique and is reported at 3% for the Bereiter technique, 9% for Dejour deepening TP and 24% for the lateral elevation technique [12]. Although the aetiology for the development of post‐operative stiffness is still unknown, various risk factors, such as complexity of the procedure, post‐operative mobilisation, and previous surgery, have been identified [10]. While this joint stiffness is well‐described following knee arthroplasty, the literature regarding patello‐femoral stabilising surgery is sparse. A systematic review on PFI surgery did not only find a lower re‐disclocation rate in the TP cohort, but a higher complication rate, with the most frequent complication being post‐operative stiffness [29].

Overall, the short‐ and mid‐term results after Bereiter TP are encouraging, with a re‐dislocation rate reported of 2%–4% and significant improvement in PROM scores [6, 19, 20, 24, 28]. In the meta‐analysis by Zaffagnini et al. [29], a mean difference of 28.1 points in the Kujala score was suggested as a clinically relevant improvement after TP with MPFL reconstruction. As shown in Table 2 the mean improvement for the Kujala score in this study is 15.6 (without AAM) and 13.0 (with AAM) after 1 year and 19 (without AAM) and 16.8 (with AAM) after 2 years; all rendered clinically irrelevant using Zaffagnini's minimal clinically important difference (MCID) suggestion. However, as stated by Leclerc et al. [19], the heterogeneity of the current literature and the variation of the outcome measures make direct comparison difficult. An MCID calculated by a distribution‐based method on the present cohort was 8.7, which is considerably lower than the improvements reported in this study, indicating the clinical relevance of improvements. Walsh et al. [27] established MCID for the International Knee Documentation Committee (IKDC), Kujala and KOOS in patients undergoing isolated MPFL‐R. While similar mean improvements, such as in the Kujala score, are observed, it is unclear if these MCIDs apply to TP patients in general or the current study population.

Subsequent surgery is considered a potential risk factor for less optimal outcomes [3, 18]. Surgery for residual PFI and cartilage degeneration may explain less optimal outcomes after TP, but the impact of AAM has not been thoroughly investigated. Wind et al. [28] reported post‐operative flexion restrictions at follow‐up in 46%, leading to re‐intervention in 13.6%, but the effects on patient‐reported outcomes were not reported. In a prospective cohort study of 62 TP patients, 11 patients (17.7%) developed arthrofibrosis [2], resulting in 9 AAMs (14.5%). However, the final ROM, PROM‐scores (Kujala, IKDC), return to work rate and satisfaction were not significantly lower for patients undergoing AAM. In the present cohort, all patients were scheduled for AAM to ensure that no intraarticular injury would occur during mechanical manipulation. Consistent with Carstensen et al., females were overrepresented, and the time to intervention was around 12 weeks. However, this study's patients had an average BMI of 22.3 points, compared to 29.9 in their study.

No guidelines for timing of AAM after TP exist, but in patients with joint stiffness after TKA intervention within the first 3 months is advocated [9]. Using a thin flap technique for TP, bone‐to‐bone healing is expected within the first 6 weeks, which is why AAM performed 3 months after Bereiter TP was considered safe.

The present study aligns with the findings of Carstensen et al. [2], showing no difference in clinical outcome could be seen 1 and 2 years after surgery between patients with and without post‐operative stiff knee. Furthermore, all patients reported statistically significant improvements in PROM scores at all follow‐up time points, and there was no difference in 2‐year improvement between patients with and without post‐operative joint stiffness. However, the post hoc power calculation reveals that the size of the population is insufficient to refute a correlation between post‐operative joint stiffness and inferior clinical outcomes.

The study is limited in that the used PROMs are not being validated for use in patients with PFI. Also, the study does not report on radiologic and clinical outcome data. In the AAM group, some analyses lacked the power to detect clinically relevant improvements; that is, they had the potential for Type II errors. However, the improvement from baseline was statistically significant and larger than 0.5 SD for all outcomes except KOOS ADL at 1‐year follow‐up, indicating a clinically relevant improvement that cannot be attributed to random variation.

## CONCLUSION

Post‐operative joint stiffness was a common complication after Bereiter TP following the Copenhagen PFI algorithm. Twenty‐five per cent of the AAM patients, or 3% of the study population, did not regain full ROM. We did not find that post‐operative joint stiffness was associated with inferior improvements in patient‐reported outcomes 1 and 2 years after surgery.

## AUTHOR CONTRIBUTIONS

Christian Dippmann and Peter Lavard conceptualised and designed the study. Christos Soleas, Anke Simone Rechter, Peter Lavard and Christian Dippmann performed the data acquisition. Volkert Siersma performed the statistical analysis and provided statistical consultation. Christian Dippmann, Kristoffer Weisskirchner Barfod and Volkert Siersma wrote and revised the manuscript. All authors reviewed.

## CONFLICT OF INTEREST STATEMENT

The authors declare no conflicts of interest.

## ETHICS STATEMENT

Ethical approval was not needed for the present study. Formal consent was given by all participating patients.

## Data Availability

All data generated or analysed during this study are included in this published article.
